# Design, Synthesis and Evaluation of Novel N-Substituted-[Benzoylamino]-5-Ethyl-1,2,3,6-Tetrahydropyridines as Potential Anti-Cancer Agents

**DOI:** 10.18689/mjpr-1000109

**Published:** 2019-03-04

**Authors:** Elizabeth D Henderson, Madhavi Gangapuram, Suresh Kumar VK Eyunni, Kinfe K Redda, Tiffany Wilson-Ardley

**Affiliations:** College of Pharmacy and Pharmaceutical Sciences, Florida A & M University, Tallahassee, FL, USA

**Keywords:** Tetrahydropyridines, Nitrogen ylides, Cancer, Antiproliferative agents, Estrogen receptor alpha

## Abstract

**Background and Objective:**

Inflammation is believed to incite carcinogenesis by causing cell and genome damage. Tetrahydropyridines have gained significant synthetic interest because they constitute biologically active features of pharmaceutical agents. Previous tetrahydropyridines developed by our research group were effective in inhibiting inflammation. Since there is a relationship between inflammation and cancer, the objective of this manuscript is to expand our prior study to determine the anti-cancer activity of novel tetrahydropyridine analogs.

**Materials and methods:**

3-Ethylpyridine reacted with O-mesitylenesulfonylhydroxylamine to furnish N-amino-3-ethylpyridinium mesitylenesulfonate. The reaction of N-amino-3-ethylpyridinium mesitylenesulfonate with substituted acid chlorides gives the stable crystalline pyridinium ylides. A sodium borohydride reduction of ylides furnishes the target compounds, N-substituted [benzoylamino]-5-ethyl-1,2,3,6-tetrahydropyridines. The evaluation of these analogs cytotoxicity against Ishikawa, MCF-7, and MDA-MB-231 cell lines were determined after 72 hours of drug exposure employing CellTiter-Glo assay. To explore the interaction between the tetrahydropyridine derivatives and estrogen receptor alpha, SYBYL-X 2.1 was used to determine the best bioactive conformations of the tetrahydropyridine derivatives for the active site of the receptor.

**Results:**

Four novel N-substituted [benzoylamino]-5-ethyl-1,2,3,6-tetrahydropyridines were synthesized, purified, and characterized. The four tetrahydropyridine analogs exhibited some anti-cancer activity. Based on the molecular modeling studies, EH3 was expected to have the best antiproliferative activity due to having the highest docking score for ERα. However, EH2 had the best antiproliferative activity. Nevertheless, the biological screening and molecular modeling can provide insight to help with the design of more biologically active compounds as potential anti-cancer agents.

## Introduction

Breast cancer is the second leading cause of cancer-related deaths in women. In 2018, the American Cancer Society estimates that 1,735,350 new cancer cases were diagnosed in the United States [1]. Approximately 80% of breast cancers are estrogen receptor positive [2]. Estrogen receptors (ER) are composed of a group of steroidal compounds named for their role in regulating menstruation and reproduction in women [3]. ER is considered a nuclear receptor that plays an important role in the growth of breast tumors. The two types of estrogen receptors are estrogen receptor alpha (ERα) and estrogen receptor beta (ERβ). ERα is mainly expressed in the mammary gland, uterus, ovary (thecal cells), bone, male reproductive organs (testes and epididymis), prostate (stroma), liver, and adipose tissue [3]. In contrast, ERβ is predominant in the prostate (epithelium), bladder, ovary (granulosa cells), colon, adipose tissue, and immune system [3]. Estrogen is a hormone produced mainly in the ovaries and is involved in breast cancer cell proliferation. Selective estrogen receptor modulators (SERMs) are various compounds that interact with intracellular estrogen receptors in different target organs as ER agonists or ER antagonists [4]. In order to inhibit the growth of breast cancer cells, antagonists (Tamoxifen) are used to block the estrogen action on tumor cells thereby preventing the binding of estrogen to the ER. Tamoxifen has been the leading drug to treat individuals that are diagnosed with ER-positive breast cancer, but unfortunately the adverse effects are problematic. Therefore, there remains a significant need to develop effective estrogen receptor modulators with less adverse effects.

Tetrahydropyridines (THPs) have gained significant synthetic interest due to possessing biological activity in natural products and pharmaceutical agents [5-10]. In previous papers, our laboratory reported the synthesis and anti-inflammatory activity of several 1,2,3,6-tetrahydropyridines [8,11-18]. Several 1,2,3,6-tetrahydropyridine derivatives have shown that their pharmacological activity depends on the position and nature of the substituents on the THP ring structure [15-17]. Due to the connection between inflammation and cancer, a series of substituted 1,3,4-oxadiazolyl tetrahydropyridines were synthesized [17]. These compounds were found to have moderate cytotoxicity *in vitro* on MCF-7 breast cancer cell lines. Due to the cytotoxicity on MCF-7 breast cancer cell lines, it is imperative to synthesize and design more tetrahydropyridine analogs that are not toxic.

It has been previously reported that a series of 5-ethyl THPs had anti-inflammatory activity [10,12]. The preliminary studies on these compounds also show some anti-cancer properties. Here, we report the anti-cancer activities of four novel 5-ethyl THP derivatives. The different substituents were chosen based on the molecular properties and bioactivity score from Molinspiration Cheminformatics [19]. The structural modifications on the THP moiety will affect the lipophilicity, size, and electron density of the compound which will also affect the activity. The molecular modeling images were generated using OpenEye Scientific Software [20]. These images provided a visual of the best conformation for the tetrahydropyridines and interaction to the active site of ERα. This analysis will provide the foundation for future optimization studies of the lead compound.

## Materials and Methods

### Synthesis of tetrahydropyridines

The structures of the ylides and tetrahydropyridines were confirmed by ^1^H-NMR, ^13^C-NMR, IR, and elemental analysis. Hydrogen nuclear magnetic resonance spectra (^1^H-NMR) were determined on a Bruker HX-300 MHz spectrometer using CDCl_3_ as solvent unless otherwise specified. Carbon nuclear magnetic resonance spectra (^13^C-NMR) were determined on a Bruker HX-600 MHz spectrometer using CDCl_3_ as solvent. Chemical shifts (δ) were reported in parts per million (ppm) downfield from trimethylsilane (TMS) as an internal standard. Fourier transform infrared spectroscopy spectra (FTIR) were ran with potassium bromide (KBr) pellets on the Perkin Elmer Spectrum 100. Elemental Analysis was performed by Atlantic Microlab, Inc. (Norcross, GA). Melting points were determined on an Electro thermal MEL-TEMP 30 melting point apparatus and were uncorrected. Chemicals and solvents were purchased by Sigma-Aldrich Chemical Company (St. Louis, MO). Purification of ylides and tetrahydropyridines were done using flash column chromatography. All reactions and purification procedures were monitored by thin layer chromatography(TLC) on Whatman 60F-245 plates AL SIL g/UV, 250 μM layer plates with visualization under ultraviolet(UV) light.

### General procedure 1

#### Synthesis of N-(1-Naphthoylimino)-3-ethylpyridinium Ylide (8a):

Ethyl acetohydroxymate (**1**) (10.23 g, 99.20 mmol) was dissolved in 50 mL of N,N-dimethylformamide at 0°C. Fifteen minutes later, mesitylene sulfonyl chloride (**2**) (21.70 g, 99.20 mmol) was slowly added along with 0.75 mL of triethylamine (Et_3_N). The solution stirred at ice bath temperature for forty-five minutes. After forty-five minutes, the reaction was poured over ice and a yellow precipitate formed. After the mixture came to room temperature, the solid, ethyl O-(mesitylenesulfonyl) acetohydroxamate (**3**) was collected by vacuum filtration. Ethyl O-(mesitylenesulfonyl) acetohydroxamate (**3**) (13.12 g, 45.98 mmol) was washed three times with 100 mL of distilled water and air dried for one hour. The product (**3**) was hydrolyzed with 70% perchloric acid (HClO_4_) (4 mL) in *p*-dioxane: water (4:1 v/v). This reaction stirred for forty-five minutes at 0°C. The reaction was arrested by the addition of ice and an off white solid, mesitylenesulfonyl hydroxamate (MSH) (**4**) [21,22] was collected by vacuum filtration and allowed to air dry for thirty minutes. MSH (9.00 g, 41.86 mmol) was dissolved in 30 mL of dichloromethane (CH_2_Cl_2_). The reaction stirred at 0°C for fifteen minutes before 3-ethylpyridine (**5**) (4.70 mL, 41.86 mmol) was added drop wise. The mixture reacted for forty-five minutes at 0°C. After forty-five minutes, the 3-ethylpyridinium mesitylenesulfonate (**6**) was extracted with 100 mL of diethyl ether.

**Figure 1.**
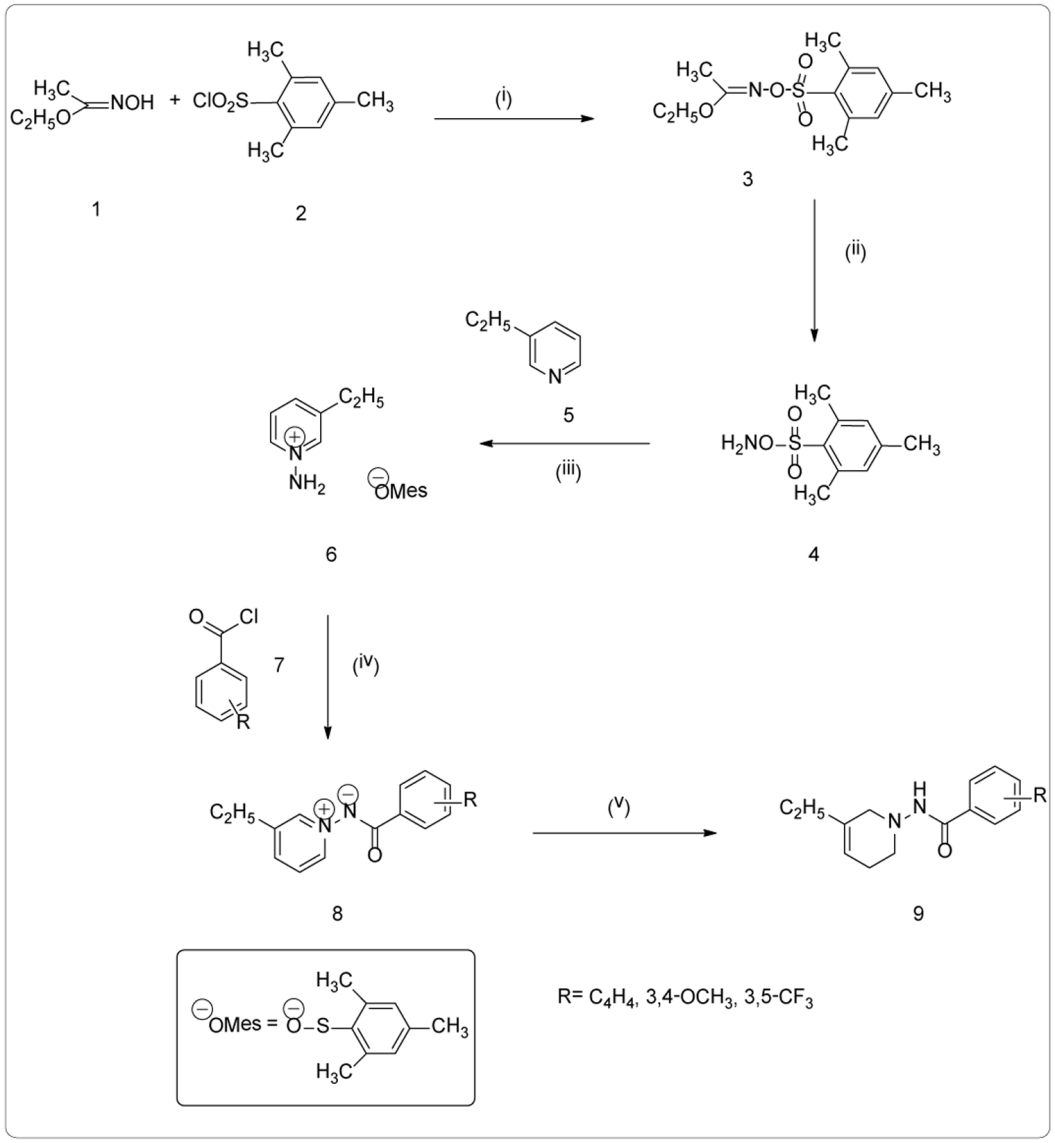
Scheme 1 Reactions and conditions: (i) DMF, Et_3_N, 0 °C, 45 min. (ii) *p*-dioxane/H_2_O, 70% HClO_4_, 45 min. (iii) 3-ethylpyridine (5), CH_2_Cl_2_, 0°C, 45 min. (iv) a.) substituted acyl chlorides (7), THF, 70°C, overnight b.) NaHCO_3_/H_2_O (v) NaBH_4_, abs. EtOH, 0°C, 7 h.

3-ethylpyridinium mesitylenesulfonate (**6**) (4.07 g, 12.56 mmol) in 20 mL of anhydrous tetrahydrofuran (THF) at 70°C was stirred for fifteen minutes before 1-naphthoyl chloride (**7**) (1.38 g, 12.56 mmol) was added. The mixture ran overnight at 70°C. After twenty-four hours, 20 mL of saturated sodium bicarbonate (NaHCO_3_) arrested the reaction. The product, N-(1-Naphthoylimino)-3-ethylpyridinium ylide (**8a**) was extracted with 100 mL of dichloromethane (CH_2_Cl_2_) three times and dried over sodium sulfate. The ylide was obtained by filtration and evaporation of the solvent, then purified by flash column chromatography using ethyl acetate: hexane (6:4 v/v). General Procedure 1 scheme is shown in figure 1. The result (**8a**) was a white solid (0.439 g, 30%); mp: 162.2-164.5°C; ^1^H-NMR (300 MHz CDCl_3_) (δ): 1.35 (t, J=7.8 Hz, 3H, CH_2_CH_3_), 2.81 (q, J=7.6 Hz, 2H, CH_2_CH_3_), 7.46-7.55 (m, 2H, C_5′_,C_9′_,-H), 7.56-7.72 (m, 1H, C_4′_-H), 7.87 (dd, J=6.9, 2.6 Hz, 2H, C_5′_, C_6′_-H), 8.19 (dd, J=8.7, 1.8 Hz, 1H, C_10′_-H), 8.26 (dd, J=8.6, 1.8 Hz, 1H, C_8′_-H), 8.68 (t, J=3.9 Hz, 2H, C_2′_, C_6_-H), 8.79 (s, 1H, C_3′_-H). *Anal.* Calcd. For C_18_H_16_N_2_O (276.33): C, 78.63; H, 5.82; N, 8.94. Found: C, 78.23; H, 5.84; N, 10.14.

#### Synthesis of N-(3,4-Dimethoxybenzoylimino)-3-ethylpyridinium Ylide (8b):

3-ethylpyridinium mesitylenesulfonate (**6**) (0.59 g, 1.82 mmol) in 30 mL of anhydrous THF at 70°C was stirred for fifteen minutes before 3,4-dimethoxybenzoyl chloride (**7**) (0.37 g, 1.82 mmol) was added. The mixture ran overnight. After twenty-four hours, 10 mL of saturated NaHCO_3_ arrested the reaction. The product, N-(3,4-dimethoxybenzoylimino)-3-ethylpyridinium ylide (**8b**) was extracted with 100 mL of CH_2_Cl_2_ three times and dried over sodium sulfate. The ylide was obtained by filtration and evaporation of the solvent, and then purified by flash column chromatography using ethyl acetate: hexane (6:4 v/v). The result (**8b**) was a white solid (0.30 g, 70%); mp: 114.3-116.9°C; ^1^H-NMR (300 MHz CDCl_3_) (δ): 1.39 (t, J=7.6 Hz, 3H, CH_2_CH_3_), 2.93 (q, J=7.6 Hz, 2H, CH_2_CH_3_), 3.92 (s, 6H, OCH_3_), 7.75 (d, J=2.1 Hz, 1H, C_5′_-H), 7.87 (dd, J=8.5, 2.1 Hz, 1H, C_5_-H), 7.95 (dd, J=8.1, 6.3 Hz, 2H, C_2′_, C_6′_-H), 8.26 (dd, J=7.4, 1.0 Hz, 1H, C_4_-H), 8.86 (d, J=7.8 Hz, 2H, C_2_, C_6_-H). *Anal.* Calcd. For C_16_H_18_N_2_O_3_ (286.33): C, 59.54; H, 6.06; N, 6.23. Found: C, 59.93; H, 5.66; N, 8.74.

#### Synthesis of N-(3,5-Trifluoromethylbenzoylimino)-3-ethylpyridinium Ylide (8c):

3-ethylpyridinium mesitylenesulfonate (**6**) (2.00 g, 6.17 mmol) in 30 mL of anhydrous THF at 70°C was stirred for fifteen minutes before 3,5-trifluoromethylbenzoyl chloride (**7**) (2.24 mL, 12.34 mmol) was added. The mixture ran overnight. After twenty-four hours, 10 mL of NaHCO_3_ arrested the reaction. The product, N-(3,5-Trifluoromethylbenzoylimino)-3-ethylpyridinium ylide (**8c**) was extracted with 100 mL of CH_2_Cl_2_ three times and dried over sodium sulfate (Na_2_SO_4_). The ylide was obtained by filtration and evaporation of the solvent, then purified by flash column chromatography using ethyl acetate: hexane (6:4 v/v). The result (**8c**) was a white solid (2.14 g, 95%); mp: 202.0-205.4°C; ^1^H-NMR (300 MHz CDCl_3_) (δ): 1.43 (t, J=7.6 Hz, 3H, CH_2_CH_3_), 2.98 (q, J=7.6 Hz, 2H, CH_2_CH_3_), 6.74 (s, 2H, C_2′_, C_6′_-H), 8.02 (dd, J=8.0, 6.3 Hz, 2H, C_5_-H), 8.32 (d, J=8.4 Hz, 1H, C_4′_-H), 8.56 (s, 2H, C_4_-H), 8.88-8.98 (m, 2H, C_2_, C_6_-H). *Anal.* Calcd. For C_16_H_12_F_6_N_2_O (362.27): C, 53.42; H, 4.28; N, 4.93. Found: C, 53.03; H, 3.34; N, 7.73.

### General procedure 2

#### Synthesis of N-(3,5-Dimethylbenzoylimino)-3-ethylpyridinium Ylide (8d):

Triethylamine (4.74 mL) was added in one portion to a suspension of N-hydroxyphthalimide (**10**) (5.00 g, 30.65 mmol) in 100 mL of acetone. The mixture was stirred at room temperature. When the reaction became a homogeneous solution, 2,4-dinitrochlorobenzene (**11**) (6.21 g, 30.65 mmol) was added and stirred at room temperature for two hours. After the two hours, a bright yellow suspension was formed and poured into 500 mL of ice water. The precipitate was filtered and washed three times with 100 mL of cold methanol (MeOH). The solid was compressed and washed three times with 100 mL of hexane and dried under vacuum to afford 2-(2,4-dinitrophenoxy)-1-H-isoindole-1,3-(2H)-dione (**12**) (7.75 g, 23.90 mmol) as an off white solid.

A solution of hydrazine hydrate (6.16 mL, 135.36 mmol) in 30 mL of MeOH was added in one portion to a solution of (**12**) in 200 mL of CH_2_Cl_2_ at 0°C. The reaction mixture rapidly became bright yellow and the precipitate was formed. The suspension was allowed to stand at 0°C for eight hours, 10 mL of cold aqueous HCl (1 N, 400 mL) was added, and the reaction was shaken rapidly at 0°C. The mixture was filtered using a Büchner funnel and the precipitate was washed three times with 50 mL of MeCN. The filtrate was poured into a separatory funnel, and the organic phase was separated. The aqueous phase was extracted twice with 100 mL of CH_2_Cl_2_. The organic phase was combined, dried over Na_2_SO_4_, filtered, and concentrated by rotoevaporation produced O-(2,4-Dinitrophenyl)hydroxylamine(**13**) [23].

**Figure 2.**
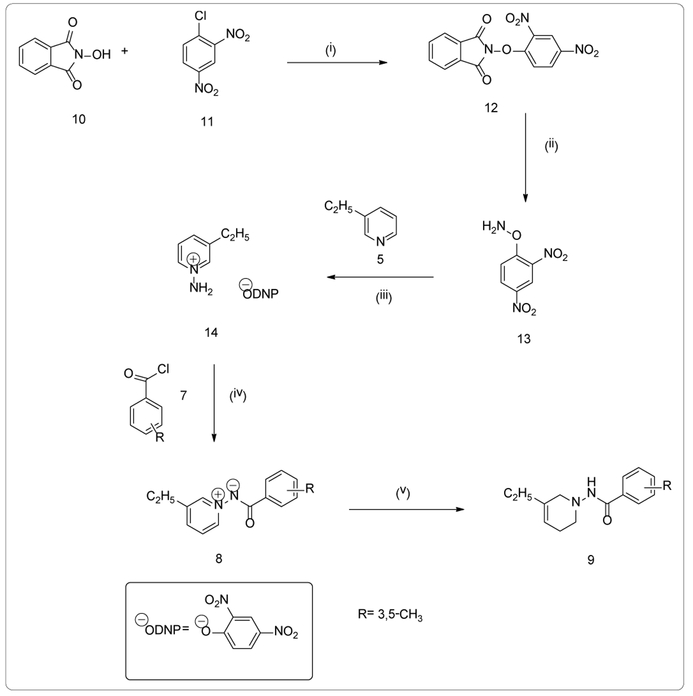
Scheme 2 reactions and conditions: (i) Et_3_N, 2,4-dinitrochlorobenzene, Acetone, RT, 2 h (ii) NH_2_NH_2_H_2_O, DCM:MeOH, 0°C, 8 h (iii) 3-ethylpyridine (5), MeCN, 40°C, 12 h (iv) a.) Substituted acyl chloride (7), THF, Et_3_N, 70°C, overnight b.) NaHCO_3_/H_2_O(v) NaBH_4_, abs. EtOH, 0°C, 7 h.

O-(2,4-Dinitrophenyl)hydroxylamine (**13**) (4.08 g, 20.53 mmol) was added to 30 mL of MeCN. 3-ethylpyridine (**5**) (2.10 mL, 18.66 mmol) was added slowly to the reaction mixture. The reaction stirred at 40°C for 12 hours. The reaction mixture turned dark red and formed the 3-ethylpyridinium 2,4-dinitrophenolate salt (**14**). The salt (**14**) (1.36 g, 4.44 mmol) was left to react with 3,5-dimethylbenzoyl chloride (**7**) (1.97 mL, 13.32 mmol) in 30 mL of THF and Et_3_N (0.93 mL, 6.66 mmol) at 70°C overnight. After twenty-four hours, 100 mL of saturated sodium bicarbonate (NaHCO_3_) arrested the reaction. The product, N-(3,5-dimethylbenzoylimino)-3-ethylpyridinium ylide (**8d**) was extracted with 100 mL of CH_2_Cl_2_ three times and dried over sodium sulfate (Na_2_SO_4_). The ylide was obtained by filtration and evaporation of the solvent, then purified by flash column chromatography using ethyl acetate: hexane (6:4 v/v). General Procedure 2 scheme is shown in figure 2. The result (**8d**) was a white solid (0.25 g, 22%); mp: 223.0-226.0°C; ^1^H-NMR (300 MHz CDCl_3_) (δ): 1.36 (t, J=7.6 Hz, 3H, CH_2_CH_3_), 2.36 (s, 6H, CH_3_), 2.91 (q, J=7.8 Hz, 2H, CH_2_CH_3_), 7.91 (s, 2H, C_2′_, C_6′_-H), 7.98 (dd, J=8.0, 6.3 Hz, 1H, C_4′_-H), 8.29 (d, J=7.8 Hz, 1H, C_5_-H), 8.79 (s, 1H, C_4_-H), 8.83 (d, J=6.3 Hz, 2H, C_2_, C_6_-H). *Anal.* Calcd. For C_16_H_18_N_2_O (254.33): C, 65.17; H, 6.40; N, 9.36. Found: C, 65.37; H, 6.17; N, 9.53.

### General procedure 3

#### Synthesis of N-(1-Naphthoylamino)-5-ethyl-1,2,3,6-tetrahydropyridines (9a):

Sodium borohydride (0.14 g, 3.60 mmol) was added to a solution of (**8a**) (0.20 g, 0.72 mmol) in 13 mL of absolute ethanol at 0°C. The reaction stirred for seven hours and was monitored by TLC. The reaction was arrested with ice and allowed to warm to room temperature. Compound (**9a**) was extracted with dichloromethane (3 × 100 mL). The combined extracts were dried over sodium sulfate (Na_2_SO_4_), filtered, and rotoevaporated. The solid obtained was purified by flash column chromatography using ethyl acetate: hexane (6:4 v/v). Rotoevaporation gave an off white solid (0.02 g, 10%); mp: 194.7- 197.5°C; IR (potassium bromide): ʋ 3161 (NH), 1661 (CO) cm^−1^; ^1^H-NMR (300 MHz CDCl_3_) (δ): 1.03 (t, J =7.5 Hz, 3H, CH_2_CH_3_), 1.98-2.04 (q, J=3, 7.8 Hz, 2H, CH_2_CH_3_), 2.35 (s, 2H, C_5_-H), 3.13 (t, J=5.9 Hz, 2H, C_2_, C_6_-H), 5.52 (s, 1H, C_4_-H), 7.26-7.59 (m, 2H, C_5′_, C_9′_-H), 7.80 (d, J=8.3 Hz, 1H, C_4′_-H), 7.90 (dd, J=15.5, 8.0 Hz, 3H, C_6′_, C_8′_, C_9′_-H), 8.27 (s, 1H, C_3′_-H), 7.01 (br, s, NH, D_2_O Exchange); ^13^C-NMR (600 MHz CDCl_3_) (δ): 11.8 (CH_2_CH_3_), 23.2 (C_5_), 27.3 (CH_2_CH_3_), 53.4 (C_6_), 56.4 (C_2_), 117.5 (C_4_), 123 (C_9′_), 127 (C_7′_), 128 (C_3′_), 129 (C_2′_), 130 (C_10′_), 132 (C_5′_), 133 (C_4′_), 134 (C_8′_), 136 (C_3_), 164 (C=O). *Anal.* Calcd. For C_18_H_20_N_2_O (280.36): C, 76.69; H, 7.25; N, 9.67. Found: C, 76.39; H, 7.12; N, 9.90.

#### Synthesis of N-(3,4-Dimethoxybenzoylamino)-5-ethyl-1,2,3,6-tetrahydropyridines (9b):

Sodium borohydride (0.13 g, 3.50 mmol) was added to a solution of (**8b**) (0.20 g, 0.70 mmol) in 13 mL of absolute ethanol at 0°C. The reaction was worked up as outlined in general procedure 3. Compound (**9b**) was extracted with dichloromethane (3 × 100 mL). The combined extracts were dried over Na_2_SO_4_, filtered, and rotoevaporated. The solid obtained was purified by flash column chromatography using ethyl acetate: hexane (6:4 v/v). Rotoevaporation gave a white solid (0.20 g, 20%); mp: 132.2-134.3°C; IR (potassium bromide): ʋ 3292 (NH), 1640 (CO) cm^−1^; ^1^H-NMR (300 MHz CDCl_3_) (δ): 1.02 (t, J=7.8 Hz, 3H, CH_2_CH_3_), 1.93-2.0 (q, J=6.9, 7.8 Hz, 2H, CH_2_CH_3_), 3.08 (t, J=5.7 Hz, 2H, C_6_-H), 3.42 (s, 2H, C_2_-H); 3.92 (s, 6H, -OCH_3_), 5.49 (s, 1H, C_4_-H), 7.23 (t, J=7.2 Hz, 1H, C_5′_-H), 7.51- 7.54 (q, J=3.0, 2.7 Hz, 1H, C_6′_-H), 7.69-7.72 (q, J=3.0 Hz, 1H, C_2′_-H), 7.01 (br, s, NH, D_2_O Exchange); ^13^C-NMR (600 MHz CDCl_3_) (δ): 11.8 (CH_2_CH_3_), 23.2 (C_4_), 27.3 (CH_2_CH_3_), 56.0 (OCH_3_), 60.3 (C_3_), 61.4 (C_2_), 111 (C_5′_), 112 (C_2′_), 117.5 (C_4_), 120.8 (C_6′_.), 127.5 (C_1′_), 136.4 (C_3_), 149 (C_3_), 153 (C_4′_), 165 (C=O). *Anal.* Calcd. For C_16_H_22_N_2_O_3_ (290.36): C, 66.18; H, 7.64; N, 9.65. Found: C, 65.97; H, 7.57; N, 9.58.

#### Synthesis of N-(3,5-Trifluoromethylbenzoylamino)-5-ethyl-1,2,3,6-tetrahydropyridines (9c):

Sodium borohydride (0.16 g, 4.15 mmol) was added to a solution of (**8c**) (0.300 g, 0.83 mmol) in 20 mL of absolute ethanol at 0°C. The reaction was worked up as outlined in general procedure 3. Compound (**9c**) was extracted with dichloromethane (3 × 100 mL). The combined extracts were dried over Na_2_SO_4_, filtered, and rotoevaporated. The solid obtained was purified by flash column chromatography using ethyl acetate: hexane (6:4 v/v). Rotoevaporation gave a white solid (0.11 g, 36 %); mp: 143.0-146.1°C; IR (potassium bromide): ʋ 3208 (NH), 1690 (CO) cm^−1^; ^1^H-NMR (300 MHz CDCl_3_) (δ): 1.03 (t, J=7.4 Hz, 3H, CH_2_CH_3_), 1.98 (q, J=8.1 Hz, 2H, CH_2_CH_3_), 3.10 (s, 2H, C_3′_, C_5′_-H), 3.44 (s, 2H, C_2_, C_6_-H), 5.48 (d, J = 18.0 Hz, 1H, C_4_-H), 8.19 (s, 2H, C_2′_, C_6′_-H), 8.34 (s, 1H, C_4_-H), 7.12 (br, s, NH, D_2_O Exchange); ^13^C-NMR (600 MHz CDCl_3_) (δ): 11.68 (CH_2_CH_3_), 23.76 (C_5_), 27.31 (CH_2_CH_3_), 52.25 (C_6_), 56.27 (C_2_), 117.31 (C_4_), 125.74 (C_4′_), 123.72 (CF_3_), 128.20 (C_2′_, C_6′_), 132.11 (C_3′_, C_5′_), 132.33 (C_1′_), 134.47 (C_3_), 163.47 (C=O). *Anal.* Calcd. For C_16_H_16_F_6_N_2_O (366.30): C, 52.46; H, 4.40; N, 7.65. Found: C, 52.70; H, 4.40; N, 7.59.

#### Synthesis of N-(3,5-Dimethylbenzoylamino)-5-ethyl-1,2,3,6-tetrahydropyridines (9d):

Sodium borohydride (0.19 g, 4.90 mmol) was added to a solution of (**8d**) (0.25 g, 0.98 mmol) in 30 mL of absolute ethanol at 0°C. The reaction was worked up as outlined in general procedure 3. Compound (**9d**) was extracted with dichloromethane (3 × 100 mL). The combined extracts were dried over Na_2_SO_4_, filtered, and rotoevaporated. The solid obtained was purified by flash column chromatography using ethyl acetate: hexane (6:4 v/v). Rotoevaporation gave a white solid (0.11 g, 44%); mp: 155.4-157.8°C; IR (potassium bromide): ʋ 3232 (NH), 1632 (CO) cm^−1^; ^1^H-NMR (300 MHz CDCl_3_) (δ): 1.00 (t, *J*=7.2 Hz, 3H, CH_2_CH_3_), 1.95 (d, *J*=6.7 Hz, 2H, CH_2_CH_3_), 2.32 (s, 6H, CH_3_), 3.17 (s, 2H, C_2_-H), 5.48 (s, 1H, C_4_-H), 7.36 (s, 2H, C_2′_, C_6′_-H), 7.11 (s, NH, D_2_O Exchange); ^13^C-NMR (600 MHz CDCl_3_) (δ): 11.77 (CH_2_CH_3_), 21.16 (CH_3_), 23.89 (C_5_), 27.40 (CH_2_CH_3_), 52.18 (C_6_), 56.36 (C_2_), 117.5 (C_4_), 126.67 (C_2′_, C_6′_), 132.00 (C_1′_), 134.07 (C_1′_), 135.05 (C_3_), 138. 51 (C_3′_), 166.13 (C=O). *Anal.* Calcd. For C_16_H_22_N_2_O (258.36): C, 74.38; H, 8.58; N, 10.84. Found: C, 74.63; H, 8.65; N, 10.76.

### Antiproliferative activity studies

Human MCF-7 and MDA-MB-231 breast cancer cell lines were purchased from the NCI. The human Ishikawa endometrial cancer cell line was purchased from Sigma Aldrich. All three cell lines were cultured in phenol red-free RPMI-1640 (Hyclone) (500 mL) supplemented with L-glutamine-dipeptide (Hyclone) (5 mL), and 10% fetal bovine serum (Atlanta Biologicals) (50 mL).

They were maintained in exponential growth phase by sub-culturing twice weekly in 150-cm^2^ flasks at 37°C, 95% air with 5% CO_2_. The media was removed from the flasks, the cells were washed with phosphate buffer solution (PBS) (HyClone) and then detached using 5 mL of TryplExpress solution (Invitrogen) (incubation 5-10 min) followed by addition of growth media. Cells were centrifuged (1,500 rpm) for 5 minutes and re-suspended in growth media at 10^5^ cells/mL. The cell lines were placed in 20, 96 well plates at a density of 5000 cells/well in total volume of 50 μL in phenol-red free medium and incubated overnight. Compounds were weighed and dissolved in DMSO (10 μM) and tested at different concentrations ranging from 0.01 to 100,000 nM. Tamoxifen (TAM, 10 μM) and 4-hydroxytamoxifen (4-OHT, 10 μM) were used as positive controls. Estradiol (25 μL of 40 nM) was added to all appropriate wells in the plate. 25 μL media was added to all wells that did not receive estradiol. 25 μL of stocks (contain compounds, DMSO and phenol-red free medium) were added to cells and medium already on plate. 50 μL media was added to media wells, 50 μL mix (contain 32 mL DMSO+768 mL phenol-red free medium) was added to all vehicle control wells and 10 μM TAM and 10 μM 4-OHT were also added to appropriate wells. Drug exposed cells were incubated for 72 hours, after which the plates were removed for CellTiter-Glo assay (Promega) from 37°C, 5% CO_2_ incubator and equilibrated at room temperature for 30 minutes.

100 μL of CellTiter-Glo assay reagent was added to each well and cell lysis was induced on an orbital shaker for 2 minutes followed by further 10 minutes incubation at room temperature. Luminescence results were read on TriLux Luminometer. The luminescent signal was proportional to the number of active cells present in culture. Dead cells do not affect cell counts because they do not contribute to ATP content. As a consequence, the number of metabolically active cells can be directly derived from the luminescent signal using a specific calibration curve. The results expressed as IC_50_ (inhibitory concentration of 50%) were the averages of three data points for each concentration and were calculated using GraphPad Prism 4.0.

### Molecular modeling

All computational studies were performed using SYBYL-X 2.1 and OpenEye Scientific software packages based on a Windows XP workstation [20]. The structures used in this manuscript were drawn using Sybyl sketch and energy minimized using MMFF94 force field and MMFF94 charges (method: Conjugate Gradient, termination: gradient 0.05 kcl/mol A°, and max. iterations: 100,000) as implemented in SYBYL. OpenEye Scientific Software’s OEDOCKING-HYBRID was the computer software used to dock the THPs to the ERα.

## Results

**Figure 3.**
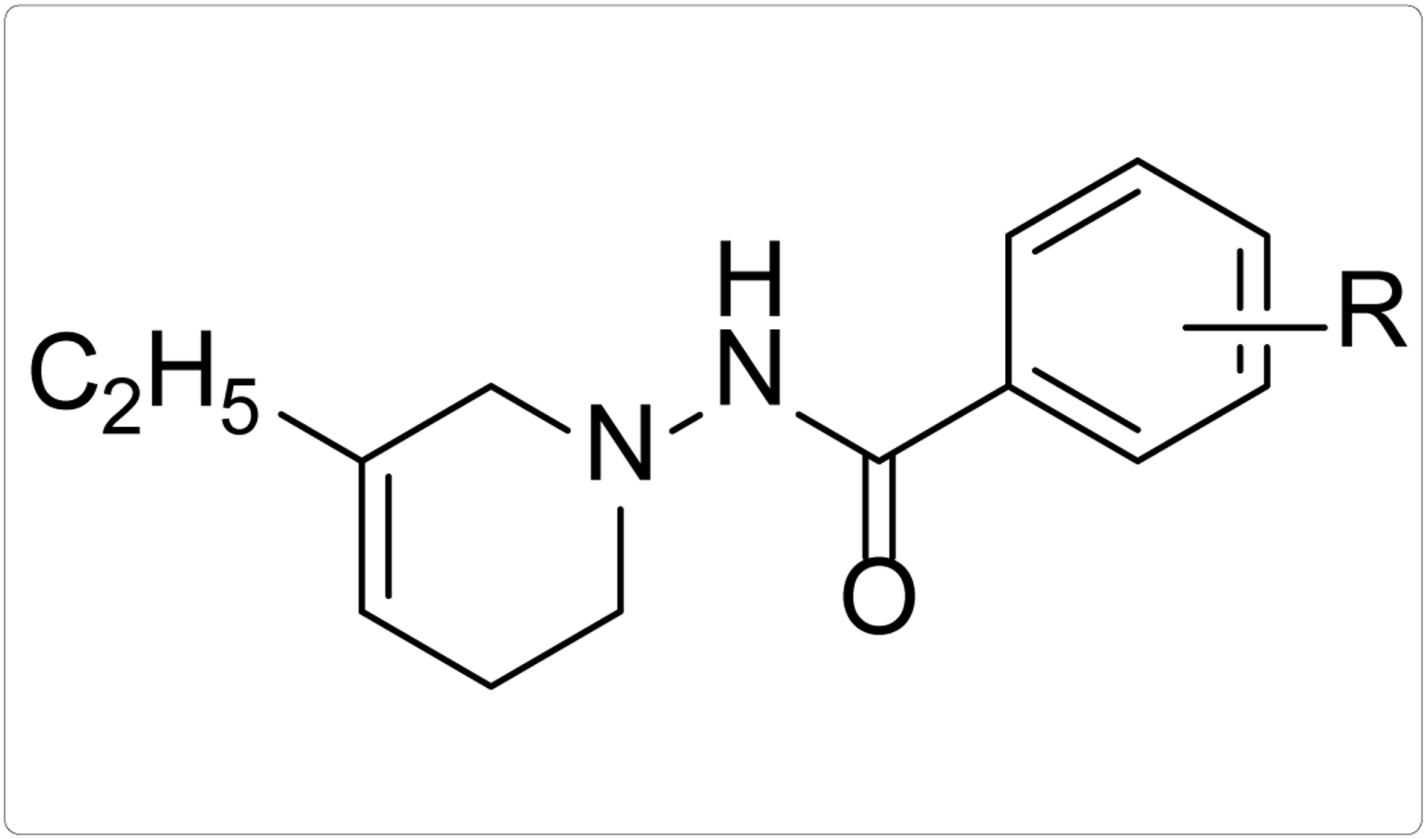
General structure of the tetrahydropyridines. R=3,4-OCH_3_, C_4_H_4_, 3,5-CF_3_, 3,5-CH_3_.

Four substituted tetrahydropyridines (Figure 3) have been synthesized, characterized, and their antiproliferative activity determined using Ishikawa, MCF-7, and MDA-MB-231 cell lines. The goal was to evaluate the effect of a few substitutions on the benzoyl ring of the THPs towards anti-cancer activity. In this regard, high-quality biological testing results were determined for the newly synthesized compounds along with Tamoxifen and 4-Hydroxytamoxifen (Table 1).

**Figure 4.**
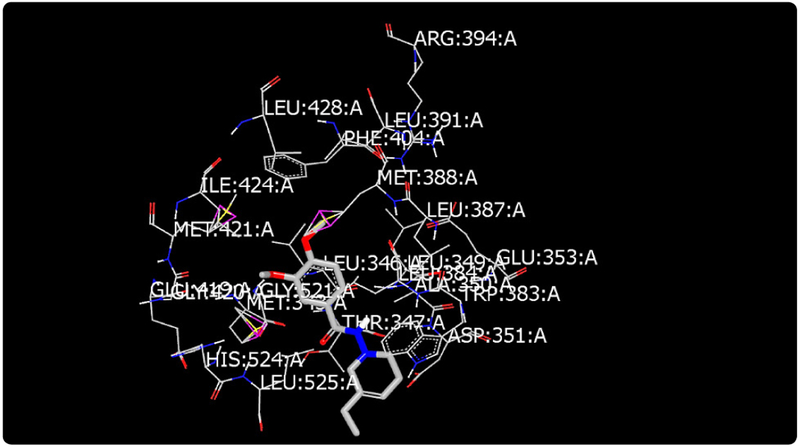
Docking of EH1 to ERα: EH1 has a slight affinity for amino acids in the active site.

**Figure 5.**
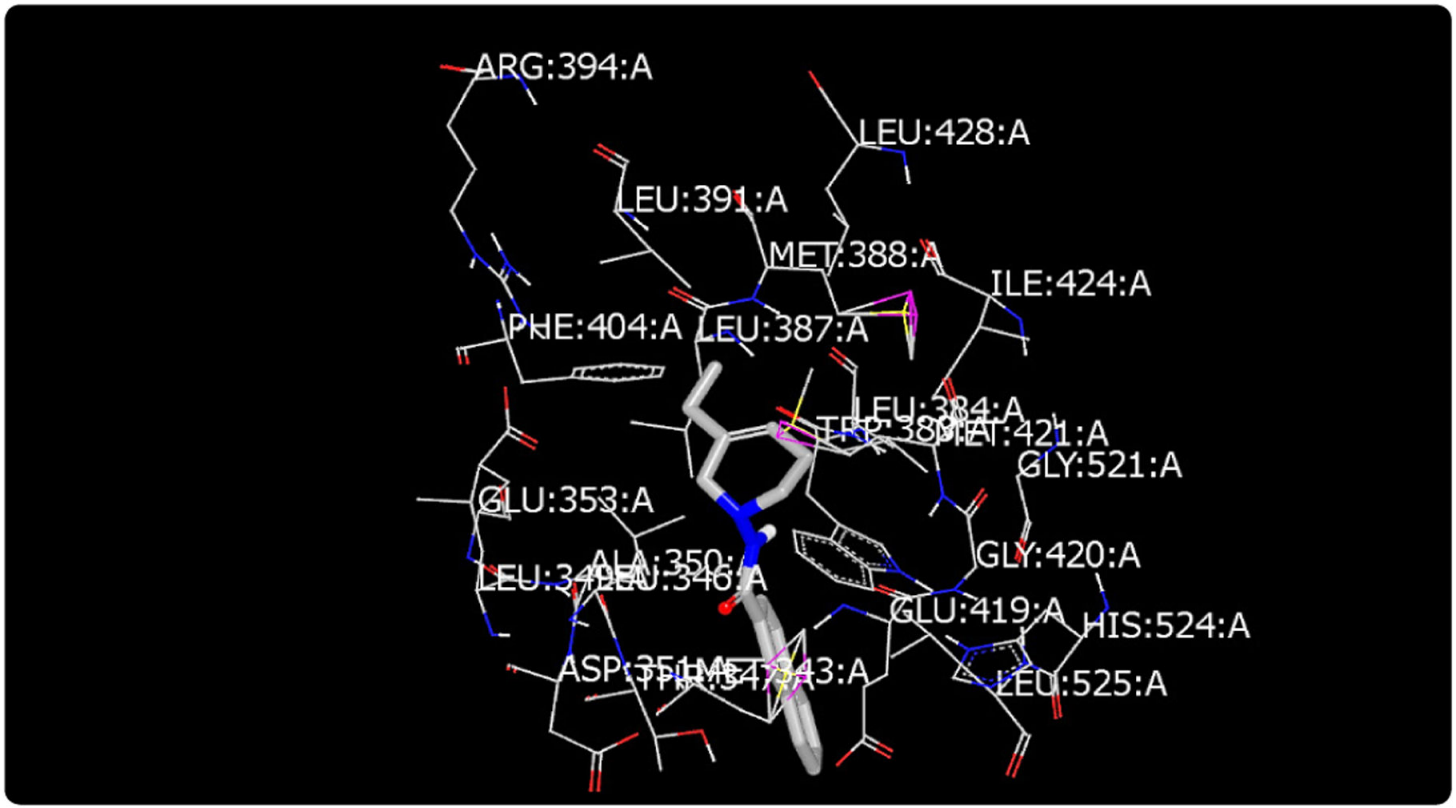
Docking of EH2 to ERα: EH2 showed pi stacking interactions with the amino acids within the active site.

**Figure 6.**
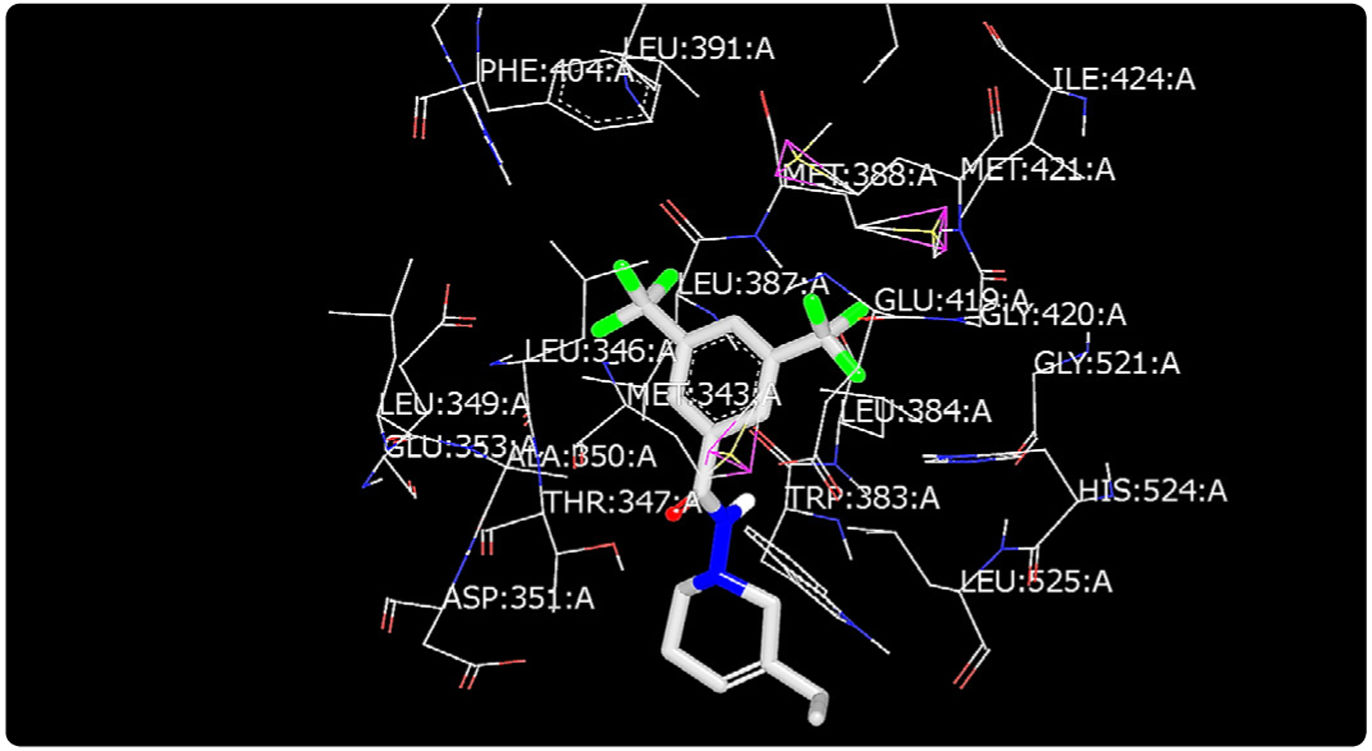
Docking of EH3 to ERα: EH3 has a slight affinity for amino acids in the active site.

**Figure 7.**
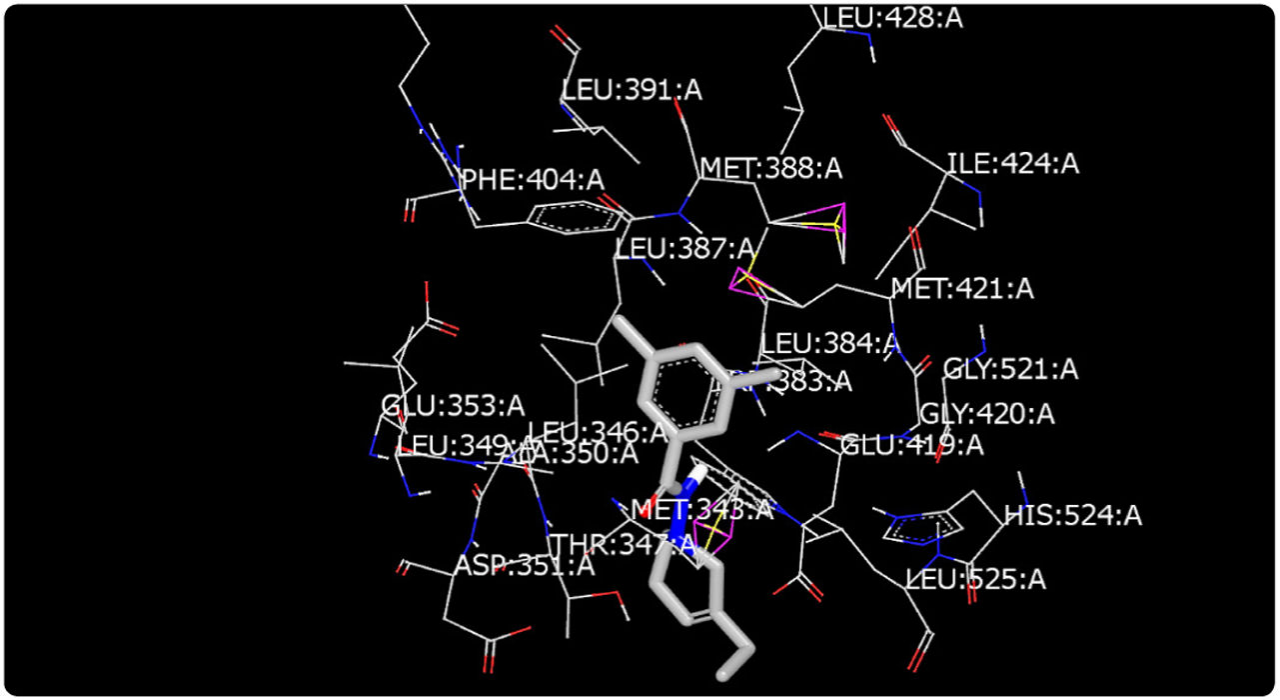
Docking of EH4 to ERα: EH4 has a slight affinity for amino acids in the active site.

Molecular modeling was used to provide an illustration of the three-dimension spatial arrangement of chemical features that are essential for biological activity. Figures 4-7 show the docking of THP derivatives to the ERα. Each figure contains the best conformation of the THPs to the active site of ERα. The docking scores for the THPs are shown in table 2. A docking score is used to predict the binding affinity between the THPs and ERα. The docking scores were calculated based on four parameters: shape, hydrogen bonding, protein desolvation, and ligand desolvation. The higher the absolute value is for the docking score the better the binding affinity is to the ERα.

## Discussion

Four novel N-Substituted [Benzoylamino]-5-ethyl-1,2,3,6-tetrahydropyridine analogs were synthesized. The antiproliferative activity of the tetrahydropyridines were conducted by Southern Research Institute. Table 1 provides the antiproliferative activities of the THPs on Ishikawa, MCF-7, and MDA-MB-231 cell lines. The IC_50_ values for Ishikawa ranged from 71.88 to greater than 100 μM for the four tetrahydropyridine derivatives. EH2 had the best IC_50_ value at 71.88 μM. EH1, EH3, and EH4 had IC_50_ values of greater than 100 μM. The IC_50_ values for MCF-7 ranged from 67.19 to greater than 100 μM for the four tetrahydropyridine derivatives. EH2 had the best IC_50_ value at 67.19 μM. EH1 and EH3 had IC_50_ values of 81.86 μM and 82.91 μM respectively. EH4 had an IC_50_ value of greater than 100 μM. The IC_50_ values for MDA-MB-231 was greater than 100 μM for all the tetrahydropyridine derivatives. The IC_50_ values for Tamoxifen and 4-Hydroxytamoxifen were 29.89 μM and 26.35 μM respectively. Although the IC_50_ values for the THPs were not as good as Tamoxifen and 4-Hydroxytamoxifen, the THP derivatives showed some antiproliferative activity.

OpenEye Scientific Software’s OEDOCKING-HYBRID was the computational software used to determine the conformation of the THPs when docked to the active site of ERα. The crystal structure of human ERα (PDB ID: 1X7E) showed that glutamic acid 353 (Glu353), arginine 394 (Arg394), glycine 521 (Gly521), and histidine 524 (His524) are the amino acid residues that play key role in stabilizing ligands embedded in the ER cavity [24]. Figures 4-7 show the docking of THP derivatives to the ERα. Each figure contains the best conformation of the THPs to the active site of ERα. Figures 4, 5, and 7 showed some hydrogen bonding and van der Waals forces. Compounds EH1, EH3, and EH4 showed a slight interaction between the amino acids that are responsible for stabilizing the ligand within the active site of ERα. Figure 5 had the most favorable conformation to the active site of ERα. Compound EH2 showed pi stacking interactions with the amino acids based on the two benzene rings on the compound. Tamoxifen’s docking scores −20.03. EH3’s docking score was −13.59. Out of the four THP derivatives, EH3 had the highest docking score. EH1’s HYBRID Chemgauss4 docking score [20] was −10.51. Out of the four THP derivatives, EH1 had the lowest docking score. EH2 and EH4’s docking scores were −10.99 and −11.02 respectively. Based on modeling, EH3 was predicted to have the best antiproliferative activity due to having the highest docking score for ERα.

## Conclusion

Four novel N-Substituted [Benzoylamino]-5-ethyl-1,2,3,6-tetrahydropyridine analogs were synthesized. The antiproliferative studies for these novel tetrahydropyridines on *Ishikawa, MCF-7,* and *MDA-MB-231* cell lines were done. EH2 had the lowest IC_50_ values for *Ishikawa* and *MCF-7* cell lines. EH3 had the highest docking score for the ERα. Based on the biological results, the docking studies did not confirm that EH3 would have the best activity. Since the THP exhibited moderate anti-cancer activities, these studies can serve as a template for synthesizing and evaluating more compounds with different structural modifications to improve the biological activities of the compounds by improving the binding affinity to ERα.

## Figures and Tables

**Table 1.** Antiproliferative Activity of Tetrahydropyridines: The antiproliferative activity studies of four tetrahydropyridines, Tamoxifen, and 4-Hydroxytamoxifen against Ishikawa, MCF-7, and MDA-MB-231 cell lines.

Compounds	Structure	IC_50_(µM)		
		ISHIKAWA	MCF-7	MDA-MB-231
EH1	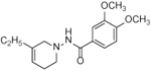	>100	81.86	>100
EH2	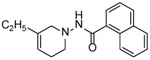	71.88	67.19	>100
EH3	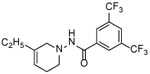	>100	82.91	>100
EH4	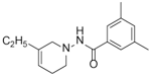	>100	>100	>100
	**Tamoxifen**	29.89	26.97	29.40
	**4-Hydroxytamoxifen**	26.35	6.78	23.07

**Table 2.** Docking Scores of THPs of ERα: The HYBRID Chemgauss4 docking scores of the four tetrahydropyridines, Tamoxifen, and 4-Hydroxytamoxifen were calculated based on four parameters. Out of the four tetrahydropyridines, EH3 had the highest docking score for ERα.

Compounds	HYBRID Chemgauss4 DOCKING SCORES
EH1	−10.51
EH2	−10.99
EH3	−13.59
EH4	−11.02
**Tamoxifen**	−**20.03**
**4-Hydroxytamoxifen**	−**18.38**

## Abbreviations

Arg: Arginine
ATP: Adenosine Triphosphate
CDCl_3_: Deuterium Chloroform
CH_2_Cl_2_: Dichloromethane
°C: Degrees Celsius
cm: centimeters
^13^C-NMR: Carbon Nuclear Magnetic Resonance
CO_2_: Carbon Dioxide
DMSO: Dimethyl Sulfoxide
ER: Estrogen Receptor
ERα: Estrogen Receptor Alpha
ERβ: Estrogen Receptor Beta
Et_3_N: Triethylamine
FTIR: Fourier Transform Infrared Spectroscopy
g: gram
Glu: Glutamic Acid
Gly: Glycine
HCl: HydrochloricAcid
HClO_4_: Perchloric Acid
His: Histidine
^1^H-NMR: Proton Nuclear Magnetic Resonance
IC_50_: Inhibitory Concentration of 50%
IR: Infrared Spectroscopy
KBr: Potassium Bromide
MeCN: Acetonitrile
MeOH: Methanol
mL: milliliters
mmol: millimole
MSH: Mesitylenesulfonyl Hydroxamate
NaHCO_3_: Sodium Bicarbonate
Na_2_SO_4_: Sodium Sulfate
nM: nanomolar
4-OHT: 4-Hydroxytamoxifen
PBS: Phosphate Buffer Solution
ppm: parts per million
RPMI: Roswell Park Memorial Institute
SERMs: Selective Estrogen Receptor Modulators
TAM: Tamoxifen
THF: Tetrahydrofuran
THP: Tetrahydropyridine
TLC: Thin Layer Chromatography
TMS: Trimethylsilane
μL: microliter
μM: micromolar
UV: Ultraviolet
δ: Chemical Shift
